# Ectopic Expression of Poplar ABC Transporter PtoABCG36 Confers Cd Tolerance in *Arabidopsis thaliana*

**DOI:** 10.3390/ijms20133293

**Published:** 2019-07-04

**Authors:** Huihong Wang, Yuanyuan Liu, Zaihui Peng, Jianchun Li, Weipeng Huang, Yan Liu, Xuening Wang, Shengli Xie, Liping Sun, Erqin Han, Nengbiao Wu, Keming Luo, Bangjun Wang

**Affiliations:** Chongqing Key Laboratory of Plant Resource Conservation and Germplasm Innovation, Key Laboratory of Eco-Environments in Three Gorges Reservoir Region (Ministry of Education), College of Life Sciences, Southwest University, Chongqing 400715, China

**Keywords:** Cd, *PtoABCG36*, tolerance, poplar, accumulation, efflux

## Abstract

Cadmium (Cd) is one of the most toxic heavy metals for plant growth in soil. ATP-binding cassette (ABC) transporters play important roles in biotic and abiotic stresses. However, few ABC transporters have been characterized in poplar. In this study, we isolated an ABC transporter gene *PtoABCG36* from *Populus tomentosa*. The *PtoABCG36* transcript can be detected in leaves, stems and roots, and the expression in the root was 3.8 and 2 times that in stems and leaves, respectively. The *PtoABCG36* expression was induced and peaked at 12 h after exposure to Cd stress. Transient expression of *PtoABCG36* in tobacco showed that PtoABCG36 is localized at the plasma membrane. When overexpressed in yeast and Arabidopsis, PtoABCG36 could decrease Cd accumulation and confer higher Cd tolerance in transgenic lines than in wild-type (WT) lines. Net Cd^2+^ efflux measurements showed a decreasing Cd uptake in transgenic Arabidopsis roots than WT. These results demonstrated that PtoABCG36 functions as a cadmium extrusion pump participating in enhancing tolerance to Cd through decreasing Cd content in plants, which provides a promising way for making heavy metal tolerant poplar by manipulating ABC transporters in cadmium polluted areas.

## 1. Introduction

Cadmium (Cd) is a highly toxic pollutant in the environment. Cadmium is nephrotoxic, and it can lead to serious human diseases, including kidney disorders, bone damage and neurotoxicity [1]. For example, high environmental exposure in Japan resulting from a stable diet of cadmium contaminated rice caused itai-itai disease [2]. Cadmium can inactivate or denature proteins by binding to the sulfhydryl groups, leading to cellular damage by displacing co-factors from a variety of proteins including transcription factors and enzymes, and by indirectly generating reactive oxygen species [3,4]. Heavy metal pollution in agricultural soils has become a serious problem. Therefore, it is essential to prevent cadmium from getting into the food chain and make the best use of cadmium contaminated soil.

Plants are able to tolerate heavy metal stress to a certain extent, with the participation of some transporters. These transporters can enhance heavy metal tolerance by pumping heavy metals into vacuoles or out of cells. Previous studies showed that two type 1(B) heavy metal-transporting subfamily of the P-type ATPases AtHMA2 and AtHMA4 are localized at the plasma membrane and can transport excessive zinc and cadmium to the outside of the cell in *Arabidopsis thaliana*, which are important players in the plant detoxification process [5]. The members of the cation diffusion facilitator (CDF) family, natural resistance-associated macrophage protein (Nramp) and Zrt/IRT-like protein (ZIP) families of transporters are also involved in the transport of heavy metals in a variety of organisms [6,7]. In addition, ATP-binding cassette (ABC) transporters are essential for plant growth and development. ABC transporters are driven by ATP hydrolysis and can act as exporters as well as importers. The Arabidopsis nuclear genome encodes for more than 100 ABC transporters, which are divided into eight subfamilies (ABCA, ABCB, ABCC, ABCD, ABCE, ABCF, ABCG and ABCI), largely exceeding that of animal. Most plant ABC transporters are present in cell membranes and are involved in detoxification processes, organ growth, plant nutrition, plant development and response to abiotic and biotic stresses [8]. Some ABC transporters are closely related to the detoxification of heavy metals. In *Saccharomyces cerevisiae*, an ABCC-like heavy metal transporter ScYCF1 (yeast cadmium factor 1) has been found to contribute to detoxifying cadmium by pumping it into vacuoles [9], and overexpression of *ScYCF1* in Arabidopsis can improve cadmium tolerance [10]. Similarly, a half-size ABC transporter HMT1 (heavy metal tolerance 1) from *Schizosaccharomyces pombe* can transport phytochelatin–Cd complexes into the vacuole, which is considered to be the first transporter to transport heavy metal-phytochelatin complexes [11]. AtABCB25/AtATM3, a close homolog of SpHMT1, contributes to Cd resistance and can transport glutamine synthetase conjugated Cd (II) across the mitochondrial membrane [12]. Full-size ABC transporters AtABCC1 and AtABCC2 have been demonstrated to be major vacuolar phytochelatins (PCs) transporters to participate in arsenic (As), mercury (Hg) and Cd resistance in Arabidopsis [13], and their homologous rice ABCC transporter OsABCC1 is involved in the As detoxification and reduces As accumulation in the rice grains [14]. Another homologous ABCC transporter PtABCC1 can enhance tolerance to Cd in poplar [15]. It has reported that AtABCC3 can complement the Cd sensitive phenotype of the *ycf1* mutant in *Saccharomyces cerevisiae* [16]. The level of expression of *AtABCC6/AtMRP6* can be up-regulated in response to cadmium (Cd) treatment [17]. Furthermore, some ABCG subfamily transporters are also involved in heavy metal resistance. AtABCG36/PDR8, localized at the plasma membrane, plays an important role in Cd extrusion from root cells [18]. The transcription of cucumber genes *CsPDR8/CsABCG36* can be up-regulated under Cd stress [19]. The rice OsABCG43/PDR5 is a Cd inducible-transporter and confers high Cd resistance in yeast cells [20].

Poplar is a woody plant with established genetic transformation system and abundant biomass. In China, there is a very large number of *Populus tomentosa*, taking up very large land resources. The previous research on poplar mainly focuses on insect resistance, herbicide resistance, biomass traits, stress tolerance, disease resistance, hormone modification, flowering modification and phytoremediation [21]. Recently, a study found that exogenous abscisic acid (ABA) stimulated the expression level of poplar ABCG40 transporter involved in lead (Pb) uptake, transport and detoxification [22]. The two multidrug and toxic compound extrusion (MATE) family genes *PtrMATE1* and *PtrMATE2* from poplar induced by aluminum (Al) can enhance aluminum resistance in acidic soils [23]. The *YCF1*-expressing transgenic poplar plants exhibited enhanced growth, reduced toxicity symptoms, and increased Cd content in the aerial tissue compared to the non-transgenic plants [24]. However, there are few studies on poplar ABCG transporters involved in Cd resistance. Therefore, the engineering of *Populus tomentosa* by manipulating ABC transporters is a significant step towards the effective utilization of Cd contaminated soil.

In the present study, we cloned a novel ABC transporter gene *PtoABCG36* (GenBank accession: MH660448) by BLAST search in the poplar database using *AtABCG36* as a query sequence. Yeast and Arabidopsis overexpressing *PtoABCG36* were measured in terms of their Cd tolerance and Cd content after Cd treatment. The results showed that overexpressing *PtoABCG36* is effective in enhancing Cd tolerance through decreasing Cd content in plants, indicating that PtoABCG36 transporter functions as a cadmium extrusion pump to participate in Cd stress in plants, which provides a reasonable way to make heavy metal tolerant poplar by manipulating ABC transporters in the areas with cadmium pollution.

## 2. Results

### 2.1. Structural and Phylogenetic Analysis of PtoABCG36

**Figure 1:**
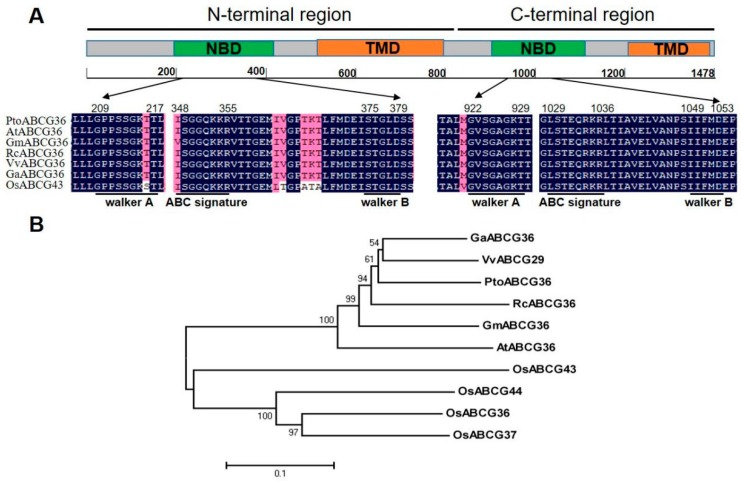
Amino acid sequence alignment and phylogenetic analysis. (**A**) Structure analysis and amino acid multi-alignment of the nucleotide-binding domains (NBD) of ABCG proteins from different plant species. ABCG domains are marked as two green and orange blocks. TMD, transmembrane domain; Walker A, ATP-binding cassette (ABC) signature; Walker B, NBD associated motifs. Blue indicates identical amino acids; pink indicates similar amino acids. (**B**) Phylogenetic analysis of ABCG proteins from *Populus trichocarpa* (PtoABCG36, MH660448); *Arabidopsis thaliana* (AtABCG36, NP_176196); *Oryza sativa* (OsABCG36, XP_015648358; OsABCG37, XP_015648329; OsABCG43, XP_015646575; OsABCG44, XP_015650488); *Glycine Max* (GmABCG36, XP_006585572); *Ricinus conmunis* (RcABCG36, XP_002515970); and *Vitis vinifera* (VvABCG29, XP_010654625); *Gossypium arboretum* (GaABCG36, XP_017606959). The numbers beside the branches represent bootstrap values based on 1000 replications.

*PtoABCG36* was isolated from full-length cDNA of leaves of six-month-old *Populus tomentosa* and submitted to GenBank (accession number: MH660448). The sequence encoded 1478 amino acid residues and contained two putative transmembrane domains (TMD) and two putative nucleotide-binding domains (NBD) (Figure 1A). Each NBD domain has about 200 amino acid residues, and it contains a Walker A motif (GXXGXGKS/T), a Walker B motif (hhhhD) and an ABC signature motif (LSGGQQ/R/KQR) [25]. Some ABCG subfamily transporters have been identified in many plant species, including *Arabidopsis thaliana*, *Glycine Max*, *Ricinus conmunis*, *Vitis vinifera*, *Gossypium arboretum* and *Oryza sativa*. The two NBD domains are highly conserved (Figure 1A).

To investigate the homology between PtoABCG36 and other plant species, ten plant ABCG transporters were analyzed. PtoABCG36 had 81.9%, 81.4%, 78.4%, 78.2% and 74.1% amino acid sequence similarity to GaABCG36 (XP_017606959), VvABCG29 (XP_010654625), GmABCG36 (XP_006585572), RcABCG36 (XP_002515970) and AtABCG36 (NP_176196), respectively. Phylogenetic analysis also revealed that PtoABCG36 was homologous with the ABCG proteins from dicotyledons such as *Vitis vinifera*, *Gossypium arboretum*, *Ricinus conmunis*, *Glycine Max* and *Arabidopsis thaliana*, as well as monocotyledons such as *Oryza sativa* (Figure 1B).

### 2.2. The PtoABCG36 Gene Is Highly Expressed in Response to Cd Stress in Poplar

**Figure 2:**
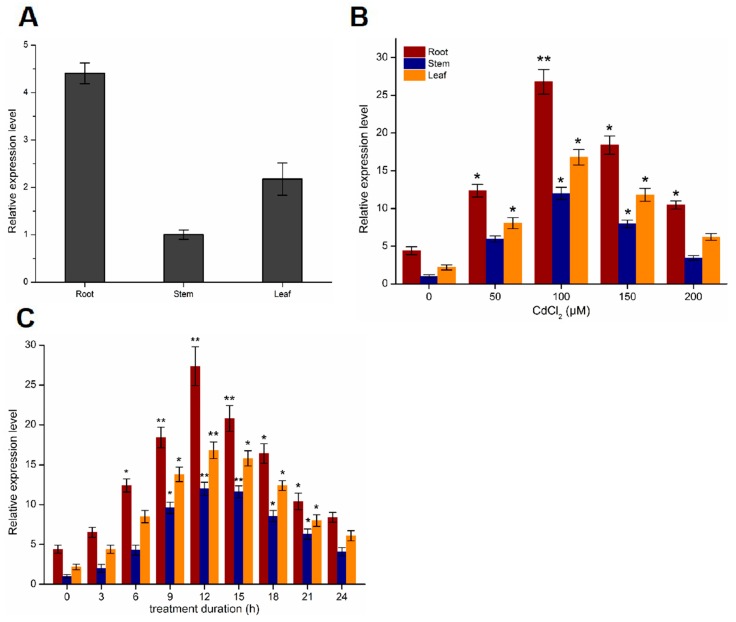
Expression analysis of *PtoABCG36* gene. (**A**) Relative expression level of *PtoABCG36* gene in roots, stems, leaves of *Populus tomentosa*. (**B**) Expression of *PtoABCG36* in poplar roots, stems and leaves under different concentrations of Cd^2+^ for12 h. (**C**) Time course of *PtoABCG36* expression in poplar roots, stems and leaves in response to 100 µM Cd^2+^ treatment. The results are shown as the mean expression ± standard deviation (SD) of three independent experiments. Poplar ubiquitin (*UBQ*) expression was used as a control and gene-specific primers were used for qRT-PCR analysis of *PtoABCG36* gene. Student’s t-test, * *p* < 0.05, ** *p* < 0.01.

To confirm the function of the PtoABCG36 transporter, we measured its gene expression level. *PtoABCG36* transcript can be detected in leaves, stems and roots, and the expression in the root was 3.8 and 2 times that of the stems and leaves, respectively. The higher expression level in the roots indicated that PtoABCG36 mainly functioned in the roots (Figure 2A). In addition, to confirm the function of PtoABCG36 in response to Cd stress, we performed induced expression using quantitative real-time PCR after the six-month-old poplars were immersed in woody plant medium (WPM) supplemented with different concentrations of CdCl_2_ for 12 h. Poplar gene-specific primers were used for qRT-PCR analysis of *PtoABCG36*. The results showed that the expression of *PtoABCG36* was significantly increased in roots with increasing cadmium concentration and reached the highest level when treated with 100 µM CdCl_2_ for 12 h. *PtoABCG36* expression was also significantly increased in stems and leaves but not as highly as that in roots. However, when treated with 150 or 200 µM CdCl_2_, the expression of the *PtoABCG36* gradually declined, but it could still be induced in roots, stems and leaves (Figure 2B). Furthermore, temporal spatial expression analysis upon treatment with 100 µM CdCl_2_ for 24 h showed that *PtoABCG36* transcript increased overtime and peaked at 12 h, with a level seven times that of the control, then gradually decreased (Figure 2C). These results further determined that *PtoABCG36* could be induced and participate in resisting Cd stress.

### 2.3. The PtoABCG36 Transporter is Localized at the Plasma Membrane

**Figure 3:**
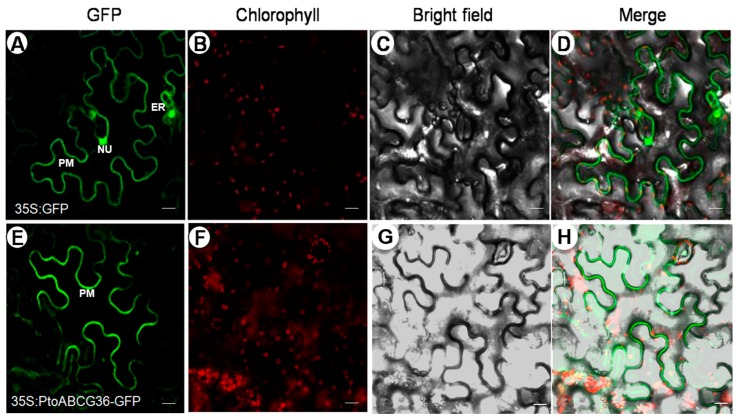
Subcellular localization of PtoABCG36 in epidermal cells of *Nicotiana benthamiana*. The fluorescence of green fluorescent protein (GFP) or PtoABCG36-GFP signal in tobacco leaf cell (**A**,**E**). Chlorophyll autofluorescence (**B**,**F**). Bright field (**C**,**G**). The overlap images of bright field and fluorescence images (**D**,**H**). Scale bars = 20 µm. NU, nucleus; PM, plasma membrane; ER, endoplasmic reticulum.

In order to determine the subcellular localization of PtoABCG36, the 35S:*PtoABCG36*-GFP construct, in which the *PtoABCG36*-GFP fusion gene was driven by the CaMV 35S promoter, was transiently expressed in the leaves of three-week-old *Nicotiana benthamiana*. Compared with the control where GFP was observed at the plasma membrane (PM), endoplasmic reticulum (ER) and nucleus (NU) in the epidermal cells (Figure 3A–D), the PtoABCG36 signal was observed only at the plasma membrane (Figure 3E–H), indicating that PtoABCG36 is localized at the plasma membrane to function as transporter, consistent with the localization pattern of AtABCG36 in *Arabidopsis thaliana*.

### 2.4. Heterologous Expression of PtoABCG36 Confers Cd Tolerance in Yeast

**Figure 4:**
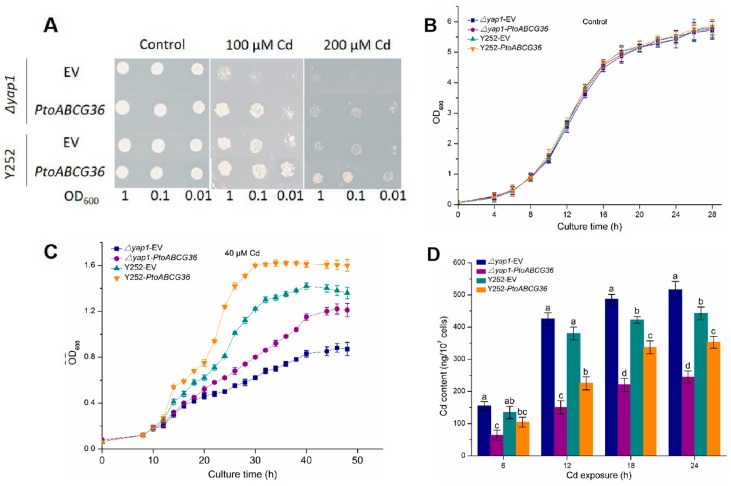
PtoABCG36 enhances cadmium tolerance in yeasts. (**A**) *Δyap1* and the wild-type Y252 were transformed with EV (pDR196 empty vector) and pDR196-*PtoABCG36*, and grown on SD plates with indicated concentrations of CdCl_2_ for 7 d. (**B**,**C**) Growth curves of yeast cells *Δyap1*-EV (square), *Δyap1- PtoABCG36* (circle), Y252- EV (up-triangle) and Y252- *PtoABCG36* (down-triangle) under control (**B**) and 40 µM CdCl_2_ condition (**C**) for indicated time. (**D**) Accumulation of cadmium in *Δyap1*-EV (navy blue), *Δyap1-PtoABCG36* (purple), Y252-EV (dark cyan) and Y252-*PtoABCG36* (orange) yeasts. Yeast cells (1 × 10^7^) were exposed to 40 µM Cd treatment for 6, 12, 18 or 24 h at 30 °C. Cd concentrations in the yeast cells were measured by ICP-OES. Error bars indicate standard deviation (*n* = 3). Different letters indicated significant differences (*p* < 0.05).

To investigate whether PtoABCG36 is involved in Cd tolerance, pDR-*PtoABCG36* was produced and transformed into the yeast Cd sensitive mutant strain *Δyap1* and wild-type strain Y252. We found that on the SD-Ura medium, growth was similar between the yeast cells carrying the empty vector and those expressing *PtoABCG36*. However, on the SD-Ura medium containing 100 µM or 200 µM CdCl_2_, the *Δyap1* or Y252 with pDR-*PtoABCG36* exhibited stronger Cd tolerance than mutants or wild-type with the empty vector (Figure 4A). Yeast growth in liquid SD-Ura medium containing 40 µM CdCl_2_ was analyzed overtime. In the absence of Cd, there was no growth difference between the *PtoABCG36*-carrying yeast and the control (Figure 4B). However, upon CdCl_2_ exposure, the growth of *PtoABCG36*-carrying *Δyap1* and Y252 were better than the yeast cells carrying the empty vector. Additionally, complementary strains partially restored their tolerance to Cd (Figure 4C), further confirming heterologous expression of *PtoABCG36* could confer Cd tolerance in yeast.

Previous studies have shown that yeast could resist cadmium by transporting it into the vacuoles or out of the cells. We tested Cd concentration in the yeast cells culturing in liquid SD-Ura medium containing 40 µM CdCl_2_. As shown in Figure 4D, after 24 h of treatment, the accumulation of Cd in *PtoABCG36*-carrying *Δyap1* and Y252 was significantly less (52.5% and 20.3% less, respectively) than that in mutant and wild-type. These results indicated that PtoABCG36 can contribute to Cd resistance by transporting it out of the yeast cells. 

### 2.5. Overexpression of PtoABCG36 Increases Tolerance to Cd and Decreases Cd Accumulation in Plants

**Figure 5:**
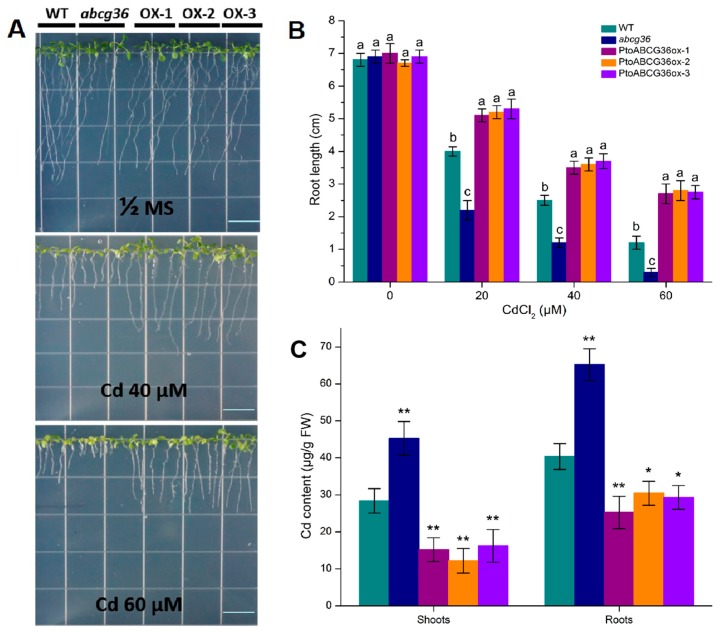
PtoABCG36 enhances cadmium tolerance in Arabidopsis. Arabidopsis seeds were grown on half-strength MS medium containing 0, 40 or 60 µM CdCl_2_ for two weeks (**A**) and primary root length (**B**) were analyzed. (**C**) Accumulation of cadmium in plants after treatment with the half MS liquid medium containing 100 µM CdCl_2_ for 24 h. For root lengths, *n* = 120–124 from three independent experiments. WT, wild-type; *abcg36*, *abcg36* mutant SALK_1422526; OX-1 and OX-2, OX-3 *PtoABCG36*-overexpressing Arabidopsis lines. White bars = 15 mm. Cd concentrations in plants were measured by ICP-OES. Error bars indicate standard deviation. Different letters indicated significant differences (*p* < 0.05). Student’s t-test, * *p* < 0.05, ** *p* < 0.01.

In order to investigate the function of PtoABCG36 in plants, the construct 35S:*PtoABCG36* was introduced into Arabidopsis. The *PtoABCG36* transcript levels were detected by qRT-PCR for further analysis (Supplementary Figure S2). Arabidopsis transgenic plants T4, wild-type and mutant seeds were analyzed after treatment without Cd and with 20 µM, 40 µM or 60 µM CdCl_2_ for 2 weeks. There was no growth difference among these lines in the absence of Cd, while the growth of Arabidopsis was significantly inhibited when grown on half MS agar plates containing 20 µM, 40 µM and 60 µM CdCl_2_. The *abcg36* mutants displayed shortest roots. However, the transgenic plants had longer roots and grew better than wild-type plants (Figure 5A,B), indicating that PtoABCG36 was also involved in mediating tolerance to Cd in plants. Quantitative analysis showed that the roots of overexpression lines (OX-2 and OX-3) were significantly longer than those of wild-type plants in the presence of 40 µM CdCl_2_ (44% and 48% longer, respectively) and 60 µM CdCl_2_ (116.7% and 112.5% longer, respectively). These results further indicated that PtoABCG36 enhanced tolerance to Cd in plants. 

To explain the detoxification mechanism of PtoABCG36 in plants, we tested the cadmium content in the mutants, wild-type and transgenic plants after treatment with the half MS liquid medium containing 100 µM CdCl_2_ for 24 h. We found that transgenic plants OX-1, OX-2 and XO-3 had lower Cd content than wild-type in the shoots (46.68%, 57% and 42.91% lower, respectively) and roots (37.42%, 22.3% and 27.37% lower, respectively). In contrast, the mutant *abcg36* had higher Cd content than the wild-type in the roots (61.65% higher) and shoots (59.24% higher). More importantly, the levels of cadmium reduction in the roots of transgenic plants were much greater than those in the shoots (Figure 5C), suggesting that PtoABCG36 contributed to Cd tolerance by pumping it out of the plants and reducing Cd toxicity in plant roots.

**Figure 6:**
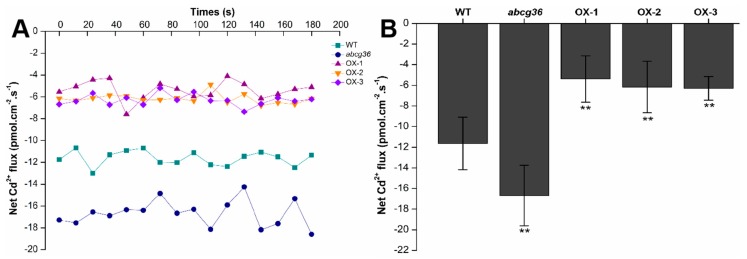
Net Cd^2+^ fluxes. Net Cd^2+^ fluxes in the roots of WT, *abcg36* mutant and transgenic plants (OX-1, OX-2, and OX-3) treated with CdCl_2_ stress (**A**). The average 180 s net Cd^2+^ fluxes are illustrated to highlight the trend differences (**B**). Bars indicate means ± SD. Student’s t-test, * *p* <0.05, ** *p* < 0.01.

To further determine the function of PtoABCG36 in plant roots, we investigated the Cd^2+^ uptake in root tips of *abcg36* mutants, WT and plants overexpressing *PtoABCG36* through a non-invasive micro-test (NMT) technique. In the presence of 50 µM CdCl_2_, the net Cd^2+^ influxes of OX-1, OX-2 and OX-3 lines were lower than WT plants (62.39%, 54.50% and 53.30% lower, respectively) (Figure 6). In contrast, the mutant *abcg36* had higher Cd net Cd^2+^ influx than the WT plants. These results indicated that a decreasing Cd uptake capacity existed in lines overexpressing *PtoABCG36* than the WT plants.

## 3. Discussion

To date, how to effectively use soil containing cadmium has become a worldwide problem. Previous studies have showed that several transporters, including the P-type ATPases AtHMA2 and AtHMA4, the CDF, Nramp and ZIP families of transporters and ABC transporters could be involved in the heavy metal tolerance [5,6,7,9,10,11].

In this study, we identified the ABC transporter ABCG36 of *Populus tomentosa.* Protein sequence analysis showed that it contained conserved Walker A, Walker B, and ABC signal (Figure 1A). In previous studies, Walker A, Walker B, ABC signal of NBD were demonstrated to function as ABC transporters motifs [26]. Phylogenetic tree analysis showed that PtoABCG36 in poplar is an ortholog of Arabidopsis AtABCG36, which acts as transporter involved in biotic or abiotic stress [18,27] (Figure 1B). Expression pattern showed that the accumulation of *PtoABCG36* transcript was mainly detected in the roots (Figure 2A). In line with our results (Figure 2B,C), it has been also reported that transcript levels of ABCG transporters were induced rapidly by biotic or abiotic stress [28,29,30,31,32]. Interestingly, *PtoABCG36* expression was induced by Cd, peaking at 12 h after Cd treatment. Additionally, the expression of *PtoABCG36* was significantly higher in poplar roots than that in shoots under Cd treatment, which is different from its ortholog in other species (Figure 2C).

It is important for plants to cope with heavy metal stress. In this study, first, we found that ectopic expression of *PtoABCG36* in yeast and Arabidopsis all significantly increased Cd tolerance (Figure 3 and Figure 5). Interestingly, our data showed that the growth of *PtoABCG36*-carrying *Δyap1* yeast stain, which has a lower level of Cd, was not better than that of the wild-type Y252 (Figure 4C,D). It is known that Yap1 increased cellular tolerance to cadmium by activating the expression of *ScYCF1* as a transcription factor. Yeast wild-type Y252 can resist Cd stress through ABC transporter ScYCF1 localized at vacuolar membrane and plasma membrane pumping Cd into vacuoles or out from the cells [10]. The expression of *YCF1* in Y252-*PtoABCG36* could pump Cd into vacuoles, while inhibition of *YCF1* in *Δyap1*-*PtoABCG36* could decrease the transport of heavy metals to vacuoles. Therefore, Y252-*PtoABCG36* has higher accumulation of Cd compared to *Δyap1*-*PtoABCG36* (Figure 4D). In addition, our data indicated that Arabidopsis *PtoABCG36*-overexpressing lines could enhance Cd tolerance (Figure 5). The *abcg36* plants are sensitive to Cd, whereas the *PtoABCG36*-overexpressing plants are tolerant (Figure 5). *PtoABCG36*-overexpressing plants have reduced cadmium content in their shoots and roots, but *abcg36* plants were the opposite. The wild-type plants accumulate 1.2 to 1.5 times as much Cd in roots and shoots as the transgenic plants (Figure 5C), suggesting that the overexpression of *PtoABCG36* could expel heavy metals from plants. Non-invasive micro-test (NMT) technique showed that overexpressing *PtoABCG36* can decrease Cd uptake capacity in plants (Figure 6). The detoxification mechanism of PtoABCG36 might be similar to that of its homologous AtABCG36 located at the plasma membrane, which can transport Cd out from the cells. 

Taken together, our study provided the evidence for the biological functions of PtoABCG36 as a transporter in regulating Cd resistance in plants. Additionally, it plays a crucial role in reducing Cd accumulation in plants, providing a theoretical basis to make heavy metal tolerant poplar by manipulating ABC transporters in cadmium polluted areas. The present study has also provided insight on the roles of ABCG transporters in economic forest cultivation. 

## 4. Materials and Methods

### 4.1. Materials and Growth Conditions

Arabidopsis seeds of wild-type (ecotype Columbia-0), *abcg36* (a loss-of-function mutant of *AtABCG36*, SALK_1422526) [18], and transgenic plants OX-1, OX-2, OX-3 were vernalized in the dark at 4 °C for 2 days, and then grew on half-strength MS agar medium plates containing 1.5% sucrose in a controlled environment with a 16 h light with 120 µmol m^−2^ s^−1^ light intensity and 8 h dark at 22 °C/18 °C for the indicated duration. 

*P. tomentosa* Carr. (clone 741) (Chinese white poplar), kindly provided by Institute of Resources Botany, Southwest University, and transgenic poplars were cultivated in a greenhouse at 24 °C under a 14 h/10 h light/dark cycle with 45 µmol m^−2^ s^−1^ of light and maintained in sterile woody plant medium (WPM) containing 0.8% (w/v) agar. Gene expression patterns were analyzed in leaves, roots and stems from 6-month-old plants.

### 4.2. Gene Cloning, Expression Vector Construction, Structural and Phylogenetic Analysis of PtoABCG36

Total RNA was extracted from the leaves of 6-month-old *P. tomentosa Carr*. by using the Trizol Reagent (Tiangen, China), then revers transcribed to cDNA by using the RT-AMV transcriptase Kit (TaKaRa, Dalian, China). The *PtoABCG36* specific fragment was amplified by PCR using specific primers (Supplementary Table S1). Cycling conditions were: 98 °C for 3 min followed by 34 cycles of 98 °C for 30 s, 56.6 °C for 30 s and 72 °C for 2 min 58 s, adding a final prolongation step at 72 °C for 10 min. The amplification products were cloned into the *Bam*HI site of the plant binary vector pCAMBIA-1300-GFP [33] as well as the *Spe*I and *Xma*I sites of the yeast vector pDR196 [34], to construct pCAMBIA-1300-*PtoABCG36* and pDR196-*PtoABCG36*.

Prediction and analysis of the structure of PtoABCG36 protein was performed with the Simple Modular Architecture Research Tool (SMART, http://smart.embl-heidelberg.de). The homologous amino acid sequences of PtoABCG36 in other species were downloaded from NCBI (http://www.ncbi.nlm.nih.gov), and aligned with DNAMAN 8.0 (Lynnon Biosoft, San Ramon, CA, USA). The phylogenetic analysis of amino acid sequences was carried out with MEGA 5.0 software by using neighbor-joining (NJ).

### 4.3. Transformation and Selection for Yeast and Arabidopsis

The yeast expression vectors pDR196 and pDR196-*PtoABCG36* were transformed into the Cd sensitive-yeast mutant *Δyap1* (*MATa ura3 lys2 ade2 trp1 leu2 yap1::leu2*) and the wild-type Y252 (*MATa ura3 lys2 ade2 trp1 leu2*) [35], kindly provided by Ji-Ming Gong (Shanghai Institutes for Biological Sciences, Chinese Academy of Sciences, shanghai, China) for metal sensitivity assay, as described [36]. Yap1 is a transcription factor that increases the tolerance of cells to cadmium by activating *YCF1* expression [37].

pCAMBIA-1300-*PtoABCG36* was transformed into the *Agrobacterium tumefaciens* strain GV3101, then transformed into wild-type Arabidopsis by the floral dip method [38]. The selection of putative transgenic plants was performed on half MS medium with 40 mg/L hygromycin and 200 mg/L cefotaxime, and further confirmed by PCR analysis (Supplementary Figure S1) and qRT-PCR analysis (Supplementary Figure S2).

### 4.4. The Metal Assay of Yeast Cells and Plants

For phenotypic analysis, yeast cells were cultured in SD-Ura liquid medium to log phase and diluted to the corresponding concentration after collection, then spotted onto SD-Ura plates containing 100 µM and 200 µM CdCl_2_. Plates were kept at 30 °C for 7 days before being photographed. Yeast cells were also cultured in liquid medium containing 40 µM CdCl_2_ for 12 h and OD_600_ was measured at indicated time [35].

For phenotypic analysis, Arabidopsis transgenic plants T4, wild-type and mutant seeds were grown on half MS agar plates in the absence or presence of 20, 40 and 60 µM CdCl_2_ for 2 weeks before being photographed and the averages of root lengths were measured in different experiments. Four untreated seedlings, each with a distinctive genotype, were grown in the half MS liquid medium with 100 µM CdCl_2_ for 24 h, and were used for determination of cadmium content. Three technical replicates were performed.

For induced expression experiment, 6-month-old poplars were immersed in WPM medium supplemented with different concentrations of CdCl_2_ for 12 h. Meanwhile, poplars treated with WPM medium without Cd were used as control. For the temporal spatial expression analysis, 6-month-old poplars were immersed in WPM medium supplemented with 100 µM CdCl_2_. Roots, stems and leaves were collected every 3 h for real-time quantitative PCR. Three technical replicates were performed.

### 4.5. Subcellular Localization of PtoABCG36

*PtoABCG36* was ligated into pCAMBIA1300-GFP vector to produce 35S:*PtoABCG36*-GFP, which was transiently expressed in the leaves of 3-week-old *Nicotiana benthamiana* to examine the subcellular localization of PtoABCG36 after 72 h of infiltration. The 35S:*PtoABCG36*-GFP construct was transformed into GV3101 cells. The cells were grown at 28 °C to OD_600_ of 0.8, resuspended in infiltration buffer (10 mM MES, pH=5.7, 10 mM MgCl_2_, and 100 µM acetosyringone) to adjust the OD_600_ to 0.6 and infiltrated into 3-week-old *Nicotiana benthamiana* leaves. Analysis was carried out with a confocal microscope (Olympus FV1200, Tokyo, Japan). Conditions for imaging were set as 488-nm excitation, collecting bandwidth at 500 to 552 nm for GFP, 633-nm excitation, collecting bandwidth at 650 to 750 nm for chlorophyll autofluorescence.

### 4.6. Quantitative Real-Time PCR Analysis

Total RNA was extracted from different plant tissues by using the RNA RNeasy Plant Mini Kit (Qiagen, Duesseldorf, Germany). First-strand cDNA synthesis was performed using the PrimeScript™ RT reagent kit (Perfect Real Time; Takara, Dalian, China). qRT-PCR was performed to detect the transcript of *PtoABCG36* in Arabidopsis and poplar by using the SYBR Green-based qPCR Master Mix (Promega, Madison, WI, USA). The gene-specific primers for qRT-PCR are listed in the supplementary Table S1. The poplar reference gene *UBQ* (FJ438462) was used as an internal control to normalize the expression data. The PCR conditions and relative gene expression calculations were conducted as previously described [14]. Three biological replicates and three technical replicates were performed.

### 4.7. Determination of Cadmium Content in Yeasts and Plants

Cells of each line (1 × 10^7^) were added to 30 mL of liquid SD-Ura medium containing 40 µM CdCl_2_ and then cultured for different durations (6, 12, 18 or 24 h) at 30 °C. The cells were then collected and washed twice with distilled water and digested with HNO_3_ and H_2_O_2_ (3:1) at 140 °C for 10 min, 200 °C for 20 min and 140 °C for 10 min. The 2-week-old plants were immersed in half MS medium supplemented with 100 µM CdCl_2_ for 24 h. Then, shoots and roots were digested with HNO_3_ and H_2_O_2_ (3:1) at 140 °C for 10 min, 200 °C for 20 min and 140 °C for 10 min [39]. All of samples were analyzed for total Cd detection by using Inductively Coupled Plasma Optical Emission Spectrometer (ICP-OES; ThermoFisher ICAP 6300, Waltham, MA, USA). All analyses were repeated three times.

### 4.8. Net Cd^2+^ Efflux Measurements

Fifteen-day-old seedlings were treated with 50 µM CdCl_2_ for 24 h and soaked in testing buffer (0.1 mM KCl, 0.1 mM CaCl_2_, 0.05 mM CdCl_2_, 0.3 mM 2-(*N*-morpholino) ethane sulfonic acid, pH 5.8) for 15 min. Roots were immobilized on the bottom of a measuring dish in fresh testing buffer. The measuring site was 800 µm from the root apex, and the net flux of Cd^2+^ was detected using a non-invasive micro-test technique (NMT; BIO-001A, Younger United States Science and Technology Corp, Beijing, China). The ion flux of Cd^2+^ was calculated according to Fick’s law of diffusion, *J*_0_ = −*D* × (*d_C_/d_X_*), where *J*_0_ is the net ion flux (in µmol·cm^−2^ per second), *D* is the self-diffusion coefficient for the ion (in cm^2^·s^−1^), *d_C_* is the difference in the ion concentrations between the two positions, and *d_X_* is the 10 µm excursion over which the electrode moved in these experiments.

### 4.9. Statistical Analysis

The experimental data related to roots length, Cd content, OD_600_ of yeast, and quantitative RT-PCR were analyzed by the statistical software SPSS 9.0. One-way analysis of variance (ANOVA) with Duncan’s multiple range tests was considered as significance test. Different letters represented significant differences (*p* < 0.05). Values represented means ± standard deviation. Quantitative difference between two groups of data for comparison in each experiment was found to be statistically significant (* *p* < 0.05; ** *p* < 0.01).

## Supplementary Materials

Supplementary materials can be found at https://www.mdpi.com/1422-0067/20/13/3293/s1.
