# Supplementary material for: Ectopic Expression of Poplar ABC Transporter PtoABCG36 Confers Cd Tolerance in Arabidopsis thaliana
Source: Int J Mol Sci. 2019 Jul 4;20(13):3293. doi: 10.3390/ijms20133293 (PMC6652139; doi:10.3390/ijms20133293)
Supplement: Supplementary file 1 [file ijms-20-03293-s001.pdf]

Supplementary Table S1

The list of primers used in PCR reactions

Gene name	Primer sequences (5′→3′)	Usage
PtoABCG36	FP-1: CGCGGATCCATGGACGGCGTAGAGAGAGCT	Primers for
	RP-1: CGCGGATCCTCTTGTCTGGAAGTTCAGTGTCC	cloning
PtoABCG36	FP-2: GGACTAGTATGGACGGCGTAGAGAGAGCT	Primers for
	RP-2: TCCCCCCGGGCTATCTTGTCTGGAAGTTCAGTG	yeast assay
PtoABCG36	FP-3: CATTACCCTATGCCATTGCAC	PCR analysis
	RP-3: TCTTGTCTGGAAGTTCAGTGTC	
PtoABCG36	FP-4: CAAGTGGTGGGTCTGGTAC	Quantitative
	RP-4: CTTGTCTGGAAGTTCAGTGTC	PCR
AtActin-1	FP-5: TGGTCGTACAACCGGTATTG	Quantitative
	RP-5: CATACTCGGCCTTGGAGATC	PCR
UBQ(FJ438462)	FP-6: GGAAGCTGCTGGTATCCACGAGAC	Quantitative
	RP-6: GCTGGAAGGTGCTGAGAGATGC	PCR

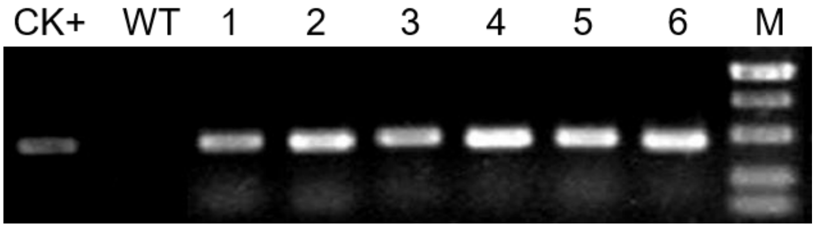

Figure S1. PCR analysis of *PtoABCG36*-overexpressing plants. Identification of *PtoABCG36* transgenic Arabidopsis plants. CK+, positive control; WT, wild type; 1-6, transgenic Arabidopsis lines; M, DNA ladder.

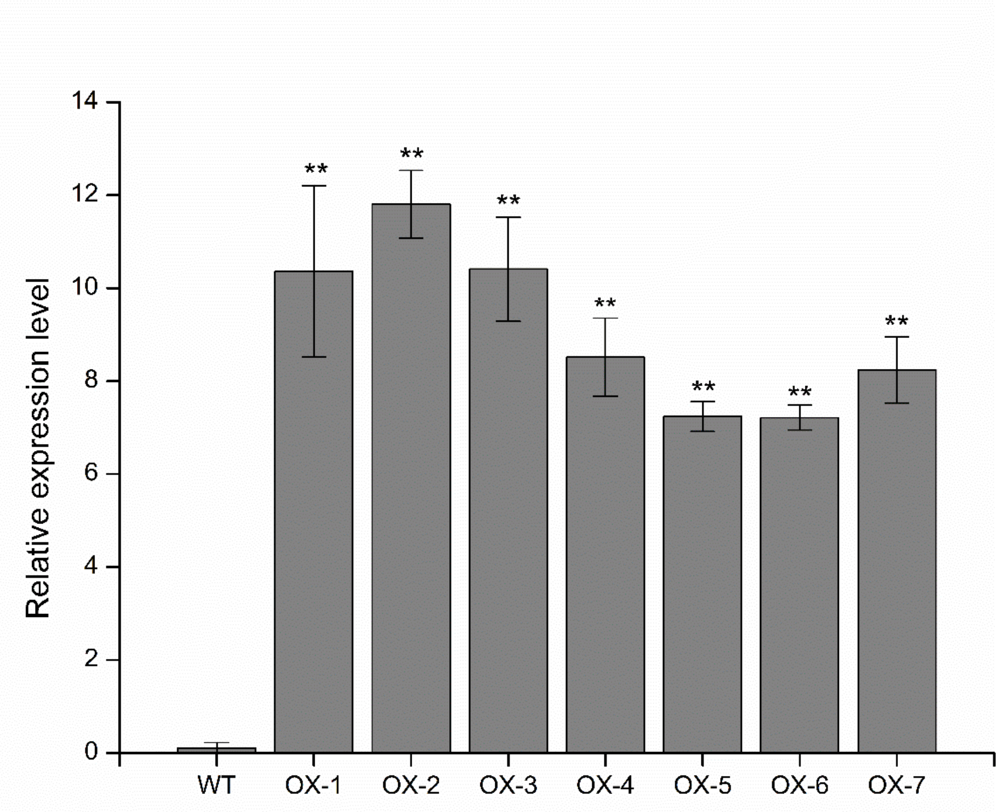

Figure S2. Relative expression level of *PtoABCG36*-overexpressing Arabidopsis plants. WT, Arabidopsis wild type; OX-1, OX-2, OX-3, OX-4, OX-5, OX-6 and OX-7, different transgenic Arabidopsis lines.
